# Impact of blood glucose on blood lactate levels in a medical ICU: a retrospective cohort study

**DOI:** 10.1186/cc10772

**Published:** 2012-03-20

**Authors:** G Adelsmayr, R Brunner, U Holzinger

**Affiliations:** 1 Medical University Vienna, Austria

## Introduction

Although blood lactate and glucose both represent important markers in the intensive care setting, they have been considered quite independently. Especially, the ideal glucose target range has been the topic of recent studies with conflicting results [1,2]. Blood lactate is an acknowledged predictor of outcome in critically ill patients [3]. The aim of this study was to establish a possible correlation between elevated blood glucose and lactate levels in intensive care patients.

## Methods

Blood gas data of 1,170 patients, admitted to the medical ICU of the Department of Medicine III, Medical University Vienna, between the years 2001 and 2009, were analysed retrospectively. The association of circulating blood glucose levels with corresponding lactate levels was investigated using a linear regression model. The impact of different blood glucose intervals (<80, 80 to 120, 120 to 160, 160 to 200, >200 mg/dl) on blood lactate levels was analysed using ANOVA. The influence of blood glucose variability, expressed as the blood glucose standard deviation, on mean lactate concentrations for the period of ICU stay was analysed using a linear regression model. To adjust for the severity of illness, a multivariate regression analysis was conducted including SAPS II and APACHE II scores.

## Results

Blood glucose and lactate presented a U-shaped curve with a minimum blood lactate (1.5 mmol/l) between 80 and 120 mg/dl blood glucose. ANOVA and linear regression demonstrated a significant influence of blood glucose and blood glucose variability on blood lactate (*P *= 0.0001). The identification of this relation was supported by the result of a multivariate regression analysis, adjusting for severity of illness (*P *= 0.0001).

## Conclusion

The results demonstrate an influence of blood glucose and blood glucose variability on blood lactate, independent of severity of illness, in a medical ICU patient population.
